# Discovery and characterization of novel small-molecule CXCR4 receptor agonists and antagonists

**DOI:** 10.1038/srep30155

**Published:** 2016-07-26

**Authors:** Rama K. Mishra, Andrew K. Shum, Leonidas C. Platanias, Richard J. Miller, Gary E. Schiltz

**Affiliations:** 1 The Center for Molecular Innovation and Drug Discovery, Northwestern University, Evanston IL, USA; 2 Department of Pharmacology, Northwestern University, Chicago IL, USA; 3 Robert H. Lurie Comprehensive Cancer Center, Feinberg School of Medicine, Northwestern University, Chicago IL, USA; 4 Department of Medicine, Jesse Brown Veterans Affairs Medical Center, Chicago IL, USA

## Abstract

The chemokine CXCL12 (SDF-1) and its cognate receptor CXCR4 are involved in a large number of physiological processes including HIV-1 infectivity, inflammation, tumorigenesis, stem cell migration, and autoimmune diseases. While previous efforts have identified a number of CXCR4 antagonists, there have been no small molecule agonists reported. Herein, we describe the identification of a novel series of CXCR4 modulators, including the first small molecules to display agonist behavior against this receptor, using a combination of structure- and ligand-based virtual screening. These agonists produce robust calcium mobilization in human melanoma cell lines which can be blocked by the CXCR4-selective antagonist AMD3100. We also demonstrate the ability of these new agonists to induce receptor internalization, ERK activation, and chemotaxis, all hallmarks of CXCR4 activation. Our results describe a new series of biologically relevant small molecules that will enable further study of the CXCR4 receptor and may contribute to the development of new therapeutics.

Although inflammatory cytokines were originally named for their important role in the regulation of immune cell function, it is now clear that they also have important effects in many other tissues including the nervous system. The “CHEMOtactic cytoKINES”, or chemokines are a case in point. These small secreted proteins exert their effects through the activation of a family of Gprotein coupled receptors (GPCRs) and were originally shown to be key mediators of the inflammatory response due to their powerful chemoattractant effects on different classes of leukocytes. However, we now know that the most ancient function of chemokine signaling concerned their ability to regulate the migration and development of stem cells. Indeed, CXCR4 chemokine receptor signaling is important in the development of all tissues^1^,^2^,^3^. For example, we previously demonstrated that SDF-1/CXCR4 was important for the formation of the hippocampal dentate gyrus (DG)^1^ and numerous other reports from our own and other laboratories have demonstrated the importance of CXCR4 signaling in the development of many structures in both the central and peripheral nervous systems^1^,^2^,^3^. Moreover, the developmental functions of CXCR4 signaling are still apparent in the adult^2^,^3^. The role of CXCR4 in anchoring hematopoietic stem cells in the bone marrow is a well-known example of this. In addition, it is also clear that CXCR4 plays an important role in the regulation of cancer metastasis^1^,^2^,^3^. Of great significance is that the CXCR4 receptor acts as a receptor for HIV-1 allowing it to infect lymphocytes and other cells^4^.

Inhibition of CXCR4 signaling may be an important therapeutic strategy in many circumstances including cancer, HIV-1 pathogenesis, and several functions within the nervous system^1^,^2^. A large number of investigations have sought to produce novel CXCR4 antagonists for therapeutic purposes^5^,^6^,^7^,^8^,^9^,^10^. In addition, CXCR4 agonists or partial agonists, which can rapidly desensitize CXCR4 receptors, might also inhibit CXCR4 signaling by such a mechanism and may also have other important signaling consequences. However, apart from peptide mimics, no small molecule CXCR4 agonists have been reported in the literature. In many cases, small molecules have advantages over peptides and proteins as molecular probes and therapeutics due to improved metabolic stability, absorption, brain penetration, and decreased immunogenicity^11^. It is therefore of great importance to develop new small molecule CXCR4 agonists and antagonists to study the biology of this receptor and to develop new therapeutics.

Previous approaches to the discovery of new CXCR4 antagonists have relied largely on ligand-based techniques because GPCRs are notoriously difficult to crystallize^12^,^13^,^14^,^15^,^16^,^17^,^18^,^19^. CXCR4 antagonists have been discovered through modification of AMD3100^7^, peptide deconstruction^8^, or high-throughput screening (HTS)^9^,^10^. Recently, several crystal structures of CXCR4 were solved that provide valuable insight into its ligand binding^20^,^21^. Analysis of the binding mode confirmed the importance of the charged residues identified from mutation studies^22^,^23^,^24^ and in addition, characterized a number of important hydrophobic interactions. Using the crystal structure with the small molecule antagonist IT1t, one group has recently published work comparing their success in virtual high-throughput screening (vHTS) using a protein homology model and the actual crystal structure^25^. Results indicated that the crystal structure provided a significantly better receptor for docking than did the model.

The above discussion indicates that the CXCR4 chemokine receptor represents an important therapeutic target for the treatment of several disorders. Herein, we report the implementation of a dual vHTS approach employing both ligand- and structure-based technique to discover a series of novel CXCR4 antagonists and agonists. These new compounds possess unprecedented CXCR4 agonist activity and are the first small molecules to do so. These compounds are important new tools in dissecting the pharmacology of CXCR4 signaling and potentially open up new avenues for therapeutics discovery against myriad diseases.

## Results

### Annotated database creation

We considered the ChemBridge GPCR-focused library containing approximately 13,000 compounds as the database for our ligand-based screen. To generate the low energy conformers, we used the ConFirm/CatConf module from the Catalyst program implemented using the “best” mode in Discovery Studio 3.1 (Discovery Studio 3.1, Accelrys, Inc. San Diego, CA). To refine the conformers, a modified version of the CHARMm force field^12^ was used along with a poling technique^13^ that biased the sampling of conformations towards geometries that were far from a local minimum but energetically near each other^14^. This method generated ~100 conformers of each compound within an energy cutoff of 10 kcal/mol.

### Common-feature pharmacophore model building and database screening

**Figure 1:**
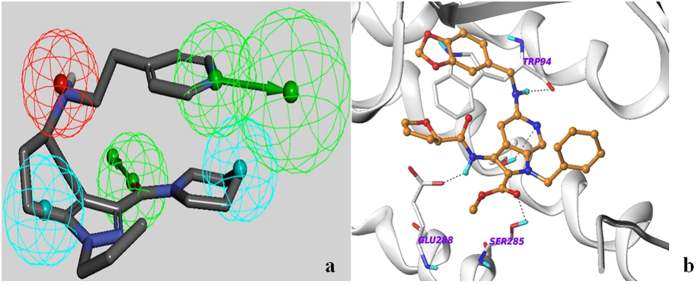
(**a**) Five-point pharmacophore used in virtual screening. Compound NUCC-390 is shown with the pharmacophore overlaid. Green = Hydrogen Bond Acceptor, Red = Positive Ionizable, Cyan = Hydrophobic. (**b**) Docked pose of NUCC-397 in the CXCR4 crystal structure. Dotted lines show putative hydrogen bonds.

Surveying ChEMBL (version 13), we selected 162 CXCR4 antagonists that were reported to have IC_50_ values in the range of 1 nM to 10 μM. Using cluster analysis protocols implemented in Discovery Studio 3.1 (Discovery Studio 3.1, Accelrys, Inc. San Diego, CA), we grouped the compounds into 5 different clusters. Since these 162 antagonists had been evaluated using different assay conditions from multiple laboratories, it was inappropriate to apply activity-based techniques. After clustering, we selected 2 molecules from each cluster to build a training set (Supplemental Table 1). We then applied the common feature pharmacophore modeling tool implemented in Discovery Studio 3.1 (Discovery Studio 3.1, Accelrys, Inc. San Diego, CA) to build a set of 10 pharmacophores, called “hypotheses”, using the training set of compounds. The default parameters were used to build the hypotheses. We then selected another 2 molecules from each of the 5 clusters (10 molecules total) to make a test set (Supplemental Table 2). All 10 hypotheses were tested with the test set and one 5-point pharmacophore model was found to fit well to all 10 compounds of the test set. This hypothesis consisted of two hydrophobic (Hy), two Hydrogen Bond Acceptor (HBA), and one Positive Ionizable (PI) feature (Fig. 1). Each of the pharmacophoric features was assigned a weight value of 1, providing a fit value of 100% for a molecule that matched all 5 features. This pharmacophore model was selected for screening the annotated GPCR compound database. Database screening produced 26 structures with >85% fit values along with conformational energy less than 5 kcal/mol. Based on availability and synthetic tractability, we purchased 6 compounds from this ligand-based hit set. Mapping of the selected pharmacophore with one of the eventual hits (NUCC-390) is shown in Fig. 1a.

### Structure-based virtual screen

Analyzing the CXCR4 crystal structures (accession codes 3ODU and 3OE0)^20^, we observed the 16-residue cyclic peptide filling a large ligand binding site, whereas the small molecule IT1t only occupies a small part of the pocket. To obtain consensus binding poses with flexible ligand docking tools, we selected two docking engines built upon orthogonal algorithms. The Surflex^26^ docking engine implemented using Sybyl-X (Sybyl-X, Certara, Inc. St. Louis, MO) and the Glide docking tool version 6.5 (Schrödinger, LLC, New York, NY) were both used as they have been found to be superior both in pose prediction and virtual screening of compound databases^27^. The Surflex docking engine is built upon a fragment-based algorithm whereas the Glide docking tool is built on a grid-based technique. Since we considered a relatively small 13,000 compound database of GPCR-focused molecules, we carried out the docking using both Surflex and Glide docking engines.

To prepare the protein for the docking experiments, the small-molecule bound CXCR4 crystal structure (pdb code 3ODU) was validated using Prime version 3.8 (Schrödinger, LLC, New York, NY) to correct for irrelevant side chains, missing atoms, undesired orientation of Asn, Gln or His residues, to replace the b-values by the OPLS charges, and to fix the protonation states of the residues at physiological pH. Next, the ‘Prot-Prep’ module was used to prepare and refine the co-crystal structure to generate the receptor (protein) and the bound ligand. A 12 Å^3^ grid box was generated using the centroid of the bound ligand to prepare for Glide docking.

For Surflex docking, the ligand (IT1t) was extracted from the co-crystal structure and the protein was subjected to the protein preparation panel in the Sybyl interface. In this panel, hydrogens were added in hydrogen bonding orientation, b-values were replaced by the Gasteiger charges, irrelevant torsions were eliminated, and the protonation states of the residues were fixed at pH 7.4. A ligand-based protomol was generated in the active site which represented the template for an ideal active-site ligand.

### Library screening

We first docked the 20 reported antagonists (Training and Test sets, see above) using the Glide-XP module with the standard sampling mode of maxkeep = 5000 and maxref = 400. The van der Waals radii for nonpolar ligand atoms were scaled to 0.8. After docking, the docked poses of the 20 compounds were analyzed and we noted the interactions of the antagonists with the different protein residues. As these compounds were of different chemotypes, they showed different binding poses interacting with different residues. The Lig-Prep module of the Schrodinger suite was utilized to prepare the GPCR-focused library for docking. Using the same docking protocols as described above, we docked the library structures into the ligand-binding site of CXCR4. Compounds showing a Glide score of <−6.0 (Schrödinger, LLC, New York, NY, 2014) were considered for further analysis. The interacting residues identified from the known antagonist set guided our analysis of the docked poses of the unknown compounds from library. Based on the docked scores and the interactions with critical residues, we selected 52 compounds from this set as *in silico* hits.

We carried out a similar approach for this docking experiment using the Surflex docking tool implemented in Sybyl interface. At first we docked the 20 antagonist set in the earlier-defined ligand-binding site of CXCR4. The default set of run time parameters had been used along with the GeomX docking mode, which generated the best docking pose of the ligands. After docking the known antagonists, we analyzed the docked poses and identified the critical interacting residues of the CXCR4 active site. Then we prepared the GPCR ligand set using the ligand preparation panel implemented in the Sybyl interface. Using similar docking protocols, we docked the library and 48 compounds showed good interactions with active site residues and had a total score >6.0 where total score is a function of −logK_d_^26^.

We found 22 compounds with similar binding poses and favorable interactions with the active site residues in common between the Glide and Surflex docking experiments. These *in silico* hits underwent further evaluation for their presence of potentially toxic or metabolically unstable groups, reactive functional groups, non-drug like features, synthetic feasibility, structural diversity, and commercial availability. Based on these criteria, 9 of these structure-based virtual hits were purchased. The docked pose of NUCC-397 along with the interacting residues is shown in Fig. 1b. Structures of all 15 compounds subjected to *in vitro* testing and their associated fit values, docking scores, and conformational energies are shown in Supplemental Table 3.

### Calcium imaging assay

**Figure 2:**
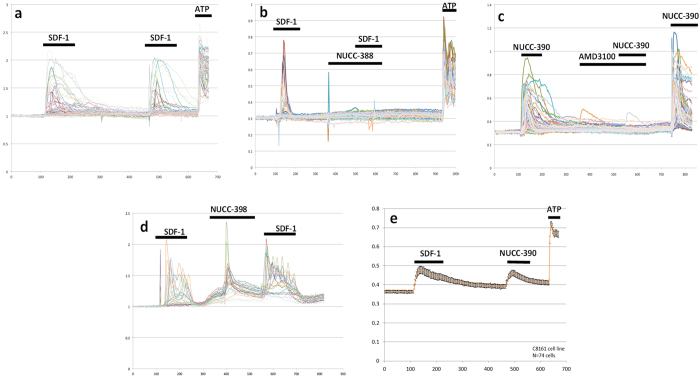
(Ca)i mobilization assay using CXCR4 expressing C8161 melanoma cells. Each colored line represents the response of a different single cell (**a**) Control using endogenous CXCR4 agonist SDF-1 (100 nM) shows two strong (Ca)i responses. Addition of ATP (10 μM) to activate purinergic receptors was performed as a positive control for cell viability (**b**) Antagonist NUCC-388 (10 μM) blocks the effect of SDF-1. (**c**) Agonist NUCC-390 (10 μM) produces strong (Ca)i response which is blocked by the known potent and selective CXCR4 antagonist AMD3100 (1 μM). (**d**) Agonist effects of NUCC-398 (10 μM). (**e**) Comparison of the effects of SDF-1 and NUCC-390 averaged over 74 cells.

Our initial assay for examining the activity of different molecules was based on the fact that activation of CXCR4 receptors produces an increase in the intracellular free Ca2^+^ concentration (Ca)i. This signal can easily be observed using a fluorescent Ca2^+^ sensing dye such as fura-2^28^,^29^,^30^. The quantitative nature of this assay makes it ideal for screening purposes. Moreover, the assay can also distinguish potential antagonists from potential agonists. We initially used the aggressive human melanoma cell line C8161 which expresses numerous human CXCR4 receptors and produces strong (Ca)i signals when stimulated with SDF-1 (Fig. 2). In the assay, cells were usually stimulated twice with SDF-1. As can be observed in Fig. 2a (control), this resulted in two (Ca)i responses of similar magnitude indicating that when applied acutely in this manner little desensitization was noted. To test a drug, the compound in question was usually added prior to the second stimulation with SDF-1. At this point it was possible to observe whether the compound itself acted as an agonist by giving its own response or if it reduced the magnitude of the second response to SDF-1. Our 15 vHTS hits were assayed at an initial single screening concentration of 10 μM and several compounds showed significant biological activity (Table 1). Some of the compounds such NUCC-388, 392, 397, and 54120 antagonized the effects of SDF-1 (Fig. 2b). Each of the four antagonists were then assayed at multiple concentrations to obtain a dose-response relationship and an estimated IC_50_. Antagonists NUCC-388, 397, 392, and 51420 had IC_50_ values of 0.3 μM, 3 μM, 1 μM, and 1 μM respectively.

Interestingly, several other molecules displayed clear agonist activity. For example, compound NUCC-390 (Fig. 2c) exhibited effects that were similar to those produced by SDF-1. The effects of NUCC-390 were clearly mediated by activation of CXCR4 receptors as they were inhibited by both AMD3100 (a highly-selective CXCR4 antagonist, Fig. 2c) and NUCC-388 (one of the novel CXCR4 antagonists, not shown) which both also blocked the effects of SDF-1. Interestingly, several other molecules in this series including NUCC-398 (Fig. 2d), 54118, 54121, and 54127 all displayed robust agonist activity when tested on C8161 cells. In each case this stimulation was demonstrated to be inhibited by AMD3100 (data not shown). Averaging data collected over a large number of cells demonstrated that the kinetics of the responses to SDF-1 and NUCC-390 were similar (Fig. 2e). In order to further demonstrate that these agonist molecules were not producing some general off-target effect, we tested some of them on the HEK 293 cell line due to its very low endogenous CXCR4 expression. We observed that SDF-1 or agonists such as NUCC-54118 and NUCC-390 produced no effect on these cells (Supplementary Figure S1).

### ERK activation by agonist NUCC-390

**Figure 3:**
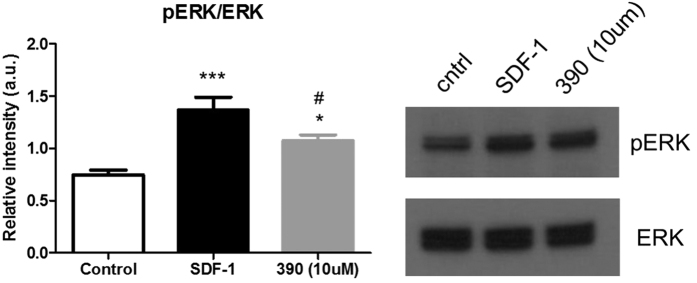
Increase in pERK produced by SDF-1 (100 nM) and NUCC-390 (10 μM) in CXCR4 expressing C8161 cells. ***p < 0.001, *p < 0.05. ^#^Different from the effect of SDF-1, p < 0.05, n = 6.

To further explore the agonist potential of compound NUCC-390, we examined changes in signaling downstream of CXCR4. For these experiments, we collected lysates from treated C8161 cells and analyzed them using Western blot. Activation of the CXCR4 receptor has been shown to indirectly mediate phosphorylation of ERK^31^,^32^, a key signaling molecule in the MAP kinase pathway. As expected, we observed that cells treated with SDF-1 for 30 min. displayed increased levels of phosphorylated ERK (pERK). Interestingly, treatment with drug NUCC-390 also led to increased levels of pERK (Fig. 3). That drug NUCC-390 has the capability of stimulating signaling activity downstream of CXCR4 receptors further supports the observation that NUCC-390 acts as a CXCR4 agonist.

### NUCC-390 induces internalization of CXCR4 receptors

**Figure 4:**
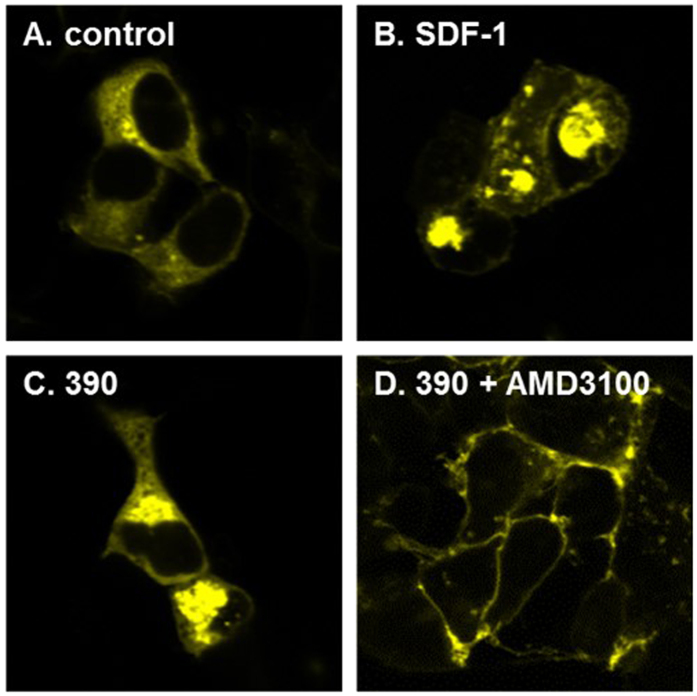
CXCR4-YFP transfected HEK293 cells treated with agonist 390. (**A**) CXCR4-YFP transfected cells show normal CXCR4 expression in the cell membrane. Pretreatment with agonist SDF-1 (100 nM) (**B**) or NUCC-390 (10 μM) (**C**) for 2 hours causes most of the CXCR4 receptor to become internalized inside cell vesicles. (**D**) Selective CXCR4 antagonist AMD3100 (1 μM) blocks internalization of agonist NUCC-390.

Another characteristic feature of CXCR4 receptors and many other GPCRs is receptor internalization following agonist stimulation^33^,^34^. In order to determine if NUCC-390 exhibited the ability to induce CXCR4 receptor internalization, we assessed the cellular localization of YFP-tagged CXCR4 receptors expressed in HEK293 cells following treatment with SDF-1 or NUCC-390. Non-treated cells showed some diffuse expression of CXCR4-YFP throughout the cytosol and clear expression in the cell membrane (Fig. 4). Treatment with SDF-1 for a period of 2 hours led to pronounced internalization of CXCR4-YFP, producing noticeable aggregates of the receptors in the cytosol but excluded from the nucleus. Similar effects were produced by NUCC-390. The effects of NUCC-390 were completely inhibited by AMD-3100 (Fig. 4d) or NUCC-388 (not shown). Interestingly, following antagonist treatment virtually all of the CXCR4-YFP was localized in the cell membrane. This might indicate some constitutive activity of the receptor and possibly inverse agonist activity for both AMD-3100 and NUCC-390^35^.

### SDF-1 and NUCC-390 mediate chemotaxis

**Figure 5:**
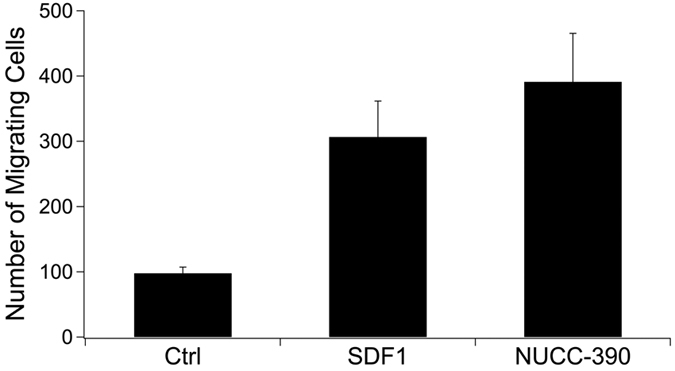
Chemotaxis produced by SDF-1 (100 nM) or NUCC-390 (10 μM) using C8161 cells in a Boyden chamber. Both SDF-1 and NUCC-390 produced significant effects (p < 0.01, n = 6).

Chemokines are well known for their ability to stimulate chemotaxis of leukocytes and stem cells. In order to further establish the biological activity of our novel CXCR4 agonists we compared the ability of SDF-1 and NUCC-390 to produce chemotaxis of C8161 cells using a Boyden chamber assay. SDF-1 produced robust chemotactic activity which was matched by the effects of NUCC-390 demonstrating that this novel agonist can produce one of the major biological effects of chemokines (Fig. 5).

### ^125^I-SDF-1α binding to the CXCR4 receptor

We assessed the interaction of NUCC-390 with CXCR4 receptors by examining the binding of ^125^I labelled SDF-1α to CXCR4 receptors in human Chem-1 cells^36^. NUCC-390 showed no significant ability to inhibit binding of ^125^I-SDF-1α to CXCR4 in concentrations up to 10^−5^ M.

## Discussion

CXCR4 receptors play a key role in the biology of stem cells in all tissues as well as in the regulation of cancer metastasis and inflammation^1^,^2^,^3^. Drugs that block CXCR4 receptors are widely used for hematopoietic stem cell transplantation and it is likely that further development of CXCR4 ligands may be useful in numerous other disorders. New CXCR4 ligands might include novel antagonists and also other types of drugs that modify the activity of GPCRs including agonists, biased agonists, inverse agonists, and positive and negative allosteric modifiers (PAMs and NAMs)^35^,^37^,^38^,^39^. Activation of CXCR4 receptors has a large number of signaling consequences for the cell including activation of diverse Gprotein and β-arrestin mediated pathways. The development of different types of agents that act on CXCR4 receptors will aid in understanding the different roles of these diverse pathways and may also constitute the basis for novel therapeutic interventions into various types of disease.

The cognate agonist for CXCR4 is the protein CXCL12 (SDF-1) which fits inside a large binding site (2049 Å^3^). This large size makes it difficult to find a small molecule that can bind in the pocket tightly, specifically, and predictably. However, mutagenesis^13^,^22^,^23^,^40^ and recent crystal structure docking studies^25^ have provided evidence of several residues that are critical for binding of small molecules. Because of these known difficulties and the relative lack of published structure-based medicinal chemistry for GPCRs, we used multiple methods for hit identification to sample as large a chemical space as possible and increase the likelihood of identifying multiple diverse drug-like CXCR4 chemotypes.

In our initial (Ca)i screen, we observed that addition of the known CXCR4-selective antagonist AMD3100 completely blocked stimulation with SDF-1, as expected. Similarly, the novel structure NUCC-388 also produced a similar blocking response. On the other hand, when compounds such as NUCC-390 and NUCC-398 were added they produced their own response indicating that they might be CXCR4 agonists. Indeed, further studies established the agonist nature of these compounds. In these studies we demonstrated that the effects of NUCC-390 and NUCC-398 could be completely inhibited by the selective CXCR4 antagonist AMD3100. The observation that molecules like NUCC-390 produce agonist effects in the absence of even small additions of SDF-1 suggest that they are orthosteric agonists rather than PAMs. Further studies will be required to determine the efficacy of each new compound and whether they are full or partial agonists. However, the observations that the effects of compounds such as NUCC-390 in the Ca imaging, ERK activation, receptor internalization, and chemotaxis assays are very similar to those produced by SDF-1, lead us to believe that they have a mostly agonist-like profile. It is also not yet clear from our experiments whether any of our new molecules are biased agonists, although the fact that they produce both Ca mobilization and receptor internalization indicates that they can access both Gprotein and β-arrestin mediated signaling pathways.

**Figure 6:**
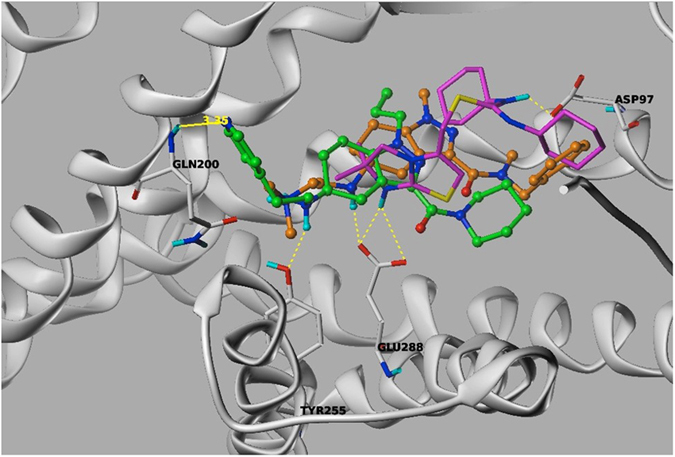
Docked pose of antagonist NUCC-388 (orange), agonist NUCC-390 (green), and antagonist ITlt from crystal structure 3ODU (magenta). Both antagonists form hydrogen bonds with Glu288 while agonist NUCC-390 does not, instead forming a hydrogen bond with Tyr255.

To rationalize how ligand binding may lead to different CXCR4 pharmacology (i.e. agonist vs. antagonist) we performed an analysis of the docked poses of NUCC-390-series antagonists and agonists presented in Table 1. Our modeling shows that the N-H group of agonist NUCC-390 and all of its analogs makes a strong hydrogen bond with Tyr255 (Fig. 6). However, both antagonists from this series (NUCC-388 and NUCC-54120) instead show strong hydrogen bonding with Glu288. Interestingly, the bound antagonist IT1t from CXCR4 crystal structure 3ODU also shows a strong hydrogen bond with Glu288 as shown in Fig. 6. From our modeling, we did not find any agonists to have a hydrogen bond interaction with Glu288.

It is interesting to note that NUCC-390 did not compete effectively with SDF-1 for binding to the CXCR4 receptor, indicating that their sites of interaction are not identical. The binding of SDF-1 is characterized by at least two separate binding pockets and it is unlikely that a molecule the size of NUCC-390 could occupy both of these. Further studies are required to fully understand the nature by which our novel agonists bind to and activate the CXCR4 receptor.

The novel molecules we have identified and studied are the first small molecule agonists of CXCR4 of which we are aware^5^. Although compounds such as NUCC-390 clearly behave as agonists *in vitro*, it will be of interest to see how they behave *in vivo* where it is possible that they will behave as *de facto* antagonists. This would be similar to the behavior of other GCPR agonists such as the S1PR agonist fingolimod, which behaves as a functional antagonist *in vivo* as a result of its ability to produce receptor internalization^41^.

In summary, we have developed a dual *in silico* screening strategy using both ligand- and structure-based approaches to identify novel small molecule modulators of the CXCR4 receptor. Testing these new compounds in a series of *in vitro* assays demonstrated agonism of the CXCR4 receptor, pharmacology never previously described for small molecules. Our lead compounds represent excellent starting points for future optimization into highly relevant probe molecules to study the function of the CXCR4 receptor in normal- and patho-physiology, and possible development as therapeutics.

## Methods

All compounds were obtained from ChemBridge and were judged to be >95% pure by analytical LC/MS as measured by UV at 254 nm. All compounds displayed a mass ion consistent with the desired structure. Calcium imaging and other assays have been previously described^28^. Compound concentrations were plotted against the percentage of cells that showed a response (defined as a Ca^2+^ transient two times above baseline). The dose-response curves for each compound were fit by the Hill equation using IGOR Pro 6.12. Estimated IC^50^/EC^50^ calculations reported were rendered from the resulting equation. CXCR4 receptor binding was performed by Eurofins using the method described^36^.

## Additional Information

**How to cite this article**: Mishra, R. K. *et al*. Discovery and characterization of novel small-molecule CXCR4 receptor agonists and antagonists. *Sci. Rep.* **6**, 30155; doi: 10.1038/srep30155 (2016).

## Supplementary Material

Supplementary Information

## Figures and Tables

**Table 1:**
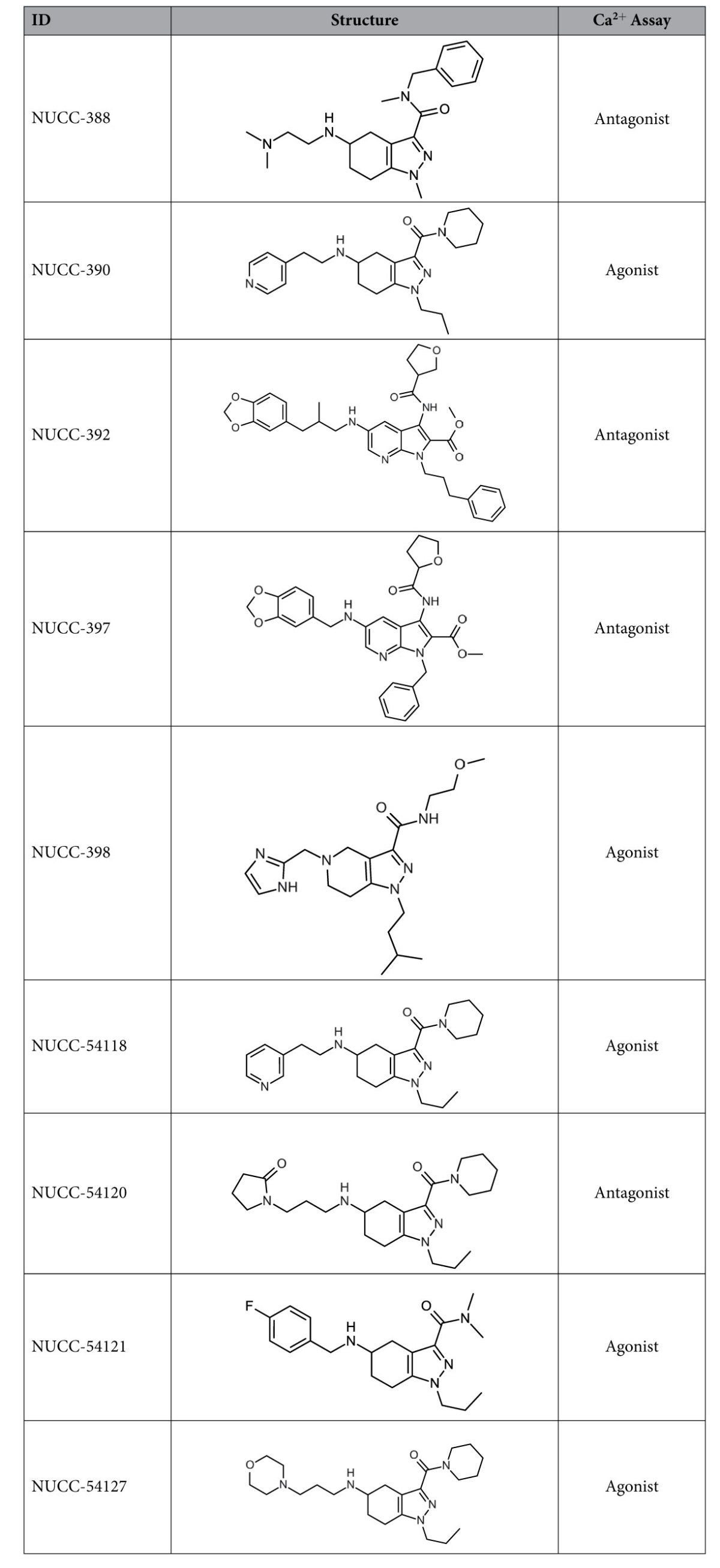
Structures and calcium imaging behavior of CXCR4 modulators.

Compounds 54118, 54120, 54121, and 54127 were close analogs of NUCC-390 that were purchased for follow-up testing after the Ca assay identified the initial hits.
