# Supplementary material for: Discovery and characterization of novel small-molecule CXCR4 receptor agonists and antagonists
Source: Sci Rep. 2016 Jul 26;6:30155. doi: 10.1038/srep30155 (PMC4960487; doi:10.1038/srep30155)
Supplement: Supplementary Information [file srep30155-s1.doc]

**Discovery and characterization of novel small-molecule CXCR4 receptor agonists and antagonists**

Rama K. Mishra,^1,4^ Andrew K. Shum,^2,4^ Leonidas C. Platanias,^3^ Richard J. Miller,^2*^ and Gary E. Schiltz^1,2*^

**AFFILIATION**

^1^The Center for Molecular Innovation and Drug Discovery, Northwestern University, Evanston IL, USA

^2^Department of Pharmacology, Northwestern University, Chicago IL, USA

^3^Robert H. Lurie Comprehensive Cancer Center and Division of Hematology-Oncology, Feinberg School of Medicine, Northwestern University, Chicago IL, USA; Department of Medicine, Jesse Brown Veterans Affairs Medical Center, Chicago IL, USA.

^4^These authors contributed equally to this work.

Supplementary Tables

| **Structure** | **Reported IC_50_ (nM)** |
| --- | --- |
|  | 1[^1^](#_ENREF_1) |
|  | 3[^2^](#_ENREF_2) |
|  | 120[^3^](#_ENREF_3) |
|  | 2200[^4^](#_ENREF_4) |
|  | 8[^5^](#_ENREF_5) |
|  | 96[^5^](#_ENREF_5) |
|  | 3200[^3^](#_ENREF_3) |
|  | 1[^6^](#_ENREF_6) |
|  | 855[^6^](#_ENREF_6) |
|  | 8120[^6^](#_ENREF_6) |
| **Supplementary Table S1**. Training set of compounds used for pharmacophore generation | |

| **Structure** | **Reported IC_50_ (nM)** |
| --- | --- |
|  | 20[^7^](#_ENREF_7) |
|  | 66[^5^](#_ENREF_5) |
|  | 1402[^3^](#_ENREF_3) |
|  | 1[^8^](#_ENREF_8) |
|  | 4800[^4^](#_ENREF_4) |
|  | 18[^5^](#_ENREF_5) |
|  | 129[^5^](#_ENREF_5) |
|  | 514[^3^](#_ENREF_3) |
|  | 173[^6^](#_ENREF_6) |
|  | 2200[^6^](#_ENREF_6) |
| **Supplementary** **Table S2**. Test set of compounds used for pharmacophore generation | |

| **ID** | **Structure** | **Docking Score (Glide)** | **Docking Score (Surflex)** | **Fit Value (Max value=5)** | **Conformational Energy Value (kcal/mol)** |
| --- | --- | --- | --- | --- | --- |
| NUCC-386 |  | N/A | N/A | 4.35 | 3 |
| NUCC-387 |  | N/A | N/A | 4.56 | 2.25 |
| NUCC-388 |  | N/A | N/A | 4.89 | 1.06 |
| NUCC-389 |  | N/A | N/A | 4.11 | 4.6 |
| NUCC-390 |  | N/A | N/A | 4.76 | 2.25 |
| NUCC-391 |  | N/A | N/A | 4.68 | 4.5 |
| NUCC-392 |  | -7.25 | 10.8 | N/A | N/A |
| NUCC-393 |  | -8.1 | 9.99 | N/A | N/A |
| NUCC-394 |  | -7.55 | 10.86 | N/A | N/A |
| NUCC-395 |  | -6.13 | 8.62 | N/A | N/A |
| NUCC-396 |  | -7.75 | 9.35 | N/A | N/A |
| NUCC-397 |  | -7.89 | 9.36 | N/A | N/A |
| NUCC-398 |  | -8.16 | 10.74 | N/A | N/A |
| NUCC-399 |  | -8.52 | 9.39 | N/A | N/A |
| NUCC-400 |  | -7.05 | 9.01 | N/A | N/A |
| **Supplementary Table S3**. Docked scores and pharmacophore fit values for *in silico* hit set. Only compounds identified in the structure-based approach will have docked scores, while only compounds identified in the ligand-based approach will have fit values. | | | | | |

**Supplementary Figures**

**
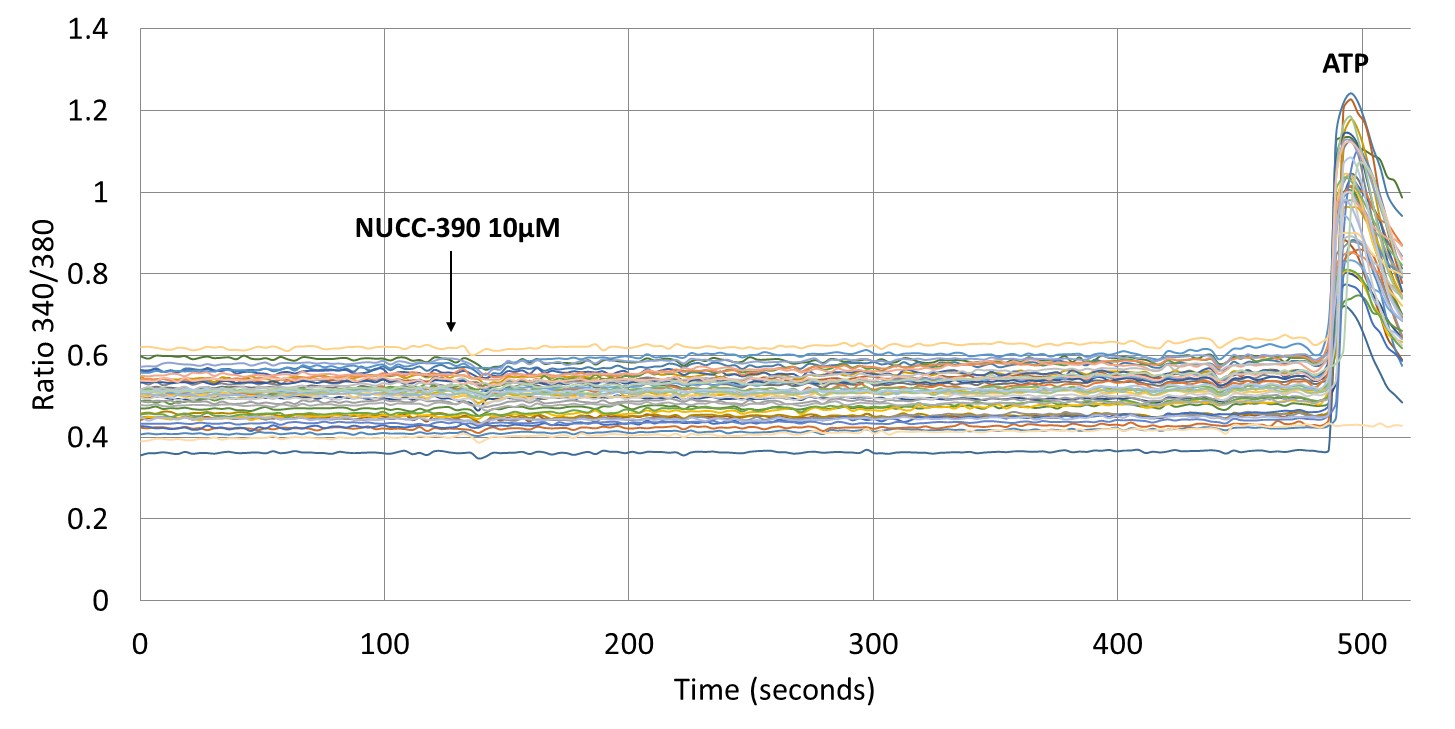
**

**Supplementary Figure S1.** (Ca)i mobilization assay using HEK293 cells. Each colored line represents the response of a different single cell. NUCC-390 (10μM) produces no off-target effects on (Ca)i in cultured HEK293 cells. Response to the purinergic receptor agonist ATP (10μM) at 500 seconds illustrates the viability of the cells.

**References**

(1) Jenkinson, S.; Thomson, M.; McCoy, D.; Edelstein, M.; Danehower, S.; Lawrence, W.; Wheelan, P.; Spaltenstein, A.; Gudmundsson, K., Blockade of X4-tropic HIV-1 cellular entry by GSK812397, a potent noncompetitive CXCR4 receptor antagonist. *Antimicrob. Agents Chemother.* **2010,** *54* (2), 817-24.

(2) Miller, J. F.; Gudmundsson, K. S.; D'Aurora Richardson, L.; Jenkinson, S.; Spaltenstein, A.; Thomson, M.; Wheelan, P., Synthesis and SAR of novel isoquinoline CXCR4 antagonists with potent anti-HIV activity. *Bioorg. Med. Chem. Lett.* **2010,** *20* (10), 3026-30.

(3) Skerlj, R. T.; Bridger, G. J.; Kaller, A.; McEachern, E. J.; Crawford, J. B.; Zhou, Y.; Atsma, B.; Langille, J.; Nan, S.; Veale, D.; Wilson, T.; Harwig, C.; Hatse, S.; Princen, K.; De Clercq, E.; Schols, D., Discovery of novel small molecule orally bioavailable C-X-C chemokine receptor 4 antagonists that are potent inhibitors of T-tropic (X4) HIV-1 replication. *J. Med. Chem.* **2010,** *53* (8), 3376-88.

(4) Ueda, S.; Kato, M.; Inuki, S.; Ohno, H.; Evans, B.; Wang, Z. X.; Peiper, S. C.; Izumi, K.; Kodama, E.; Matsuoka, M.; Nagasawa, H.; Oishi, S.; Fujii, N., Identification of novel non-peptide CXCR4 antagonists by ligand-based design approach. *Bioorg. Med. Chem. Lett.* **2008,** *18* (14), 4124-9.

(5) Skerlj, R.; Bridger, G.; McEachern, E.; Harwig, C.; Smith, C.; Kaller, A.; Veale, D.; Yee, H.; Skupinska, K.; Wauthy, R.; Wang, L.; Baird, I.; Zhu, Y.; Burrage, K.; Yang, W.; Sartori, M.; Huskens, D.; De Clercq, E.; Schols, D., Design of novel CXCR4 antagonists that are potent inhibitors of T-tropic (X4) HIV-1 replication. *Bioorg. Med. Chem. Lett.* **2011,** *21* (5), 1414-8.

(6) Thoma, G.; Streiff, M. B.; Kovarik, J.; Glickman, F.; Wagner, T.; Beerli, C.; Zerwes, H. G., Orally bioavailable isothioureas block function of the chemokine receptor CXCR4 in vitro and in vivo. *J. Med. Chem.* **2008,** *51* (24), 7915-20.

(7) Skerlj, R.; Bridger, G.; McEachern, E.; Harwig, C.; Smith, C.; Wilson, T.; Veale, D.; Yee, H.; Crawford, J.; Skupinska, K.; Wauthy, R.; Yang, W.; Zhu, Y.; Bogucki, D.; Di Fluri, M.; Langille, J.; Huskens, D.; De Clercq, E.; Schols, D., Synthesis and SAR of novel CXCR4 antagonists that are potent inhibitors of T tropic (X4) HIV-1 replication. *Bioorg. Med. Chem. Lett.* **2011,** *21* (1), 262-6.

(8) Murakami, T.; Kumakura, S.; Yamazaki, T.; Tanaka, R.; Hamatake, M.; Okuma, K.; Huang, W.; Toma, J.; Komano, J.; Yanaka, M.; Tanaka, Y.; Yamamoto, N., The novel CXCR4 antagonist KRH-3955 is an orally bioavailable and extremely potent inhibitor of human immunodeficiency virus type 1 infection: comparative studies with AMD3100. *Antimicrob. Agents Chemother.* **2009,** *53* (7), 2940-8.
